# An Implantable Wireless Neural Interface System for Simultaneous Recording and Stimulation of Peripheral Nerve with a Single Cuff Electrode

**DOI:** 10.3390/s18010001

**Published:** 2017-12-21

**Authors:** Ahnsei Shon, Jun-Uk Chu, Jiuk Jung, Hyungmin Kim, Inchan Youn

**Affiliations:** 1 Daegu Research Center for Medical Devices and Rehabilitation Engineering, Korea Institute of Machinery and Materials, 330, Techno Sunhwan-ro, Yuga-myeon, Dalseong-gun, Daegu 42994, Korea; ahnsei@kimm.re.kr (A.S.); jijoung@kimm.re.kr (J.J.); 2 Biomedical Research Institute, Korea Institute of Science and Technology, 5, Hwarang-ro 14-gil, Seongbuk-gu, Seoul 02792, Korea; hk@kist.re.kr

**Keywords:** electroneurogram, implantable medical device, MICS-band-based radio link, nerve cuff electrode, wireless power transmission

## Abstract

Recently, implantable devices have become widely used in neural prostheses because they eliminate endemic drawbacks of conventional percutaneous neural interface systems. However, there are still several issues to be considered: low-efficiency wireless power transmission; wireless data communication over restricted operating distance with high power consumption; and limited functionality, working either as a neural signal recorder or as a stimulator. To overcome these issues, we suggest a novel implantable wireless neural interface system for simultaneous neural signal recording and stimulation using a single cuff electrode. By using widely available commercial off-the-shelf (COTS) components, an easily reconfigurable implantable wireless neural interface system was implemented into one compact module. The implantable device includes a wireless power consortium (WPC)-compliant power transmission circuit, a medical implant communication service (MICS)-band-based radio link and a cuff-electrode path controller for simultaneous neural signal recording and stimulation. During in vivo experiments with rabbit models, the implantable device successfully recorded and stimulated the tibial and peroneal nerves while communicating with the external device. The proposed system can be modified for various implantable medical devices, especially such as closed-loop control based implantable neural prostheses requiring neural signal recording and stimulation at the same time.

## 1. Introduction

Neural prosthetic devices have been used for clinical purposes in various applications, including foot-drop correction [1], handgrip assistance [2], pain relief [3] and bladder control [4]. In the past, those kind of neural interface devices that use percutaneous connections between their electrodes and external devices not only restrict the subject’s mobility but also carry a serious risk of infection because of the lead wires penetrating the skin. In addition, the tethered cables are subject to considerable wear and often contaminate the neural signals due to external noise caused by the subject’s movements and nearby power lines [5,6,7]. To overcome these drawbacks, implantable wireless devices have attracted significant attention in recent years due to the elimination of infection and noise produced by the wires [8,9,10,11,12]. Although these devices have resolved the problems caused by connected wires, certain drawbacks still remain, including low-efficiency power transmission, short-range data communication with high power consumption and limited functionality (i.e., acting either as a neural signal recorder or as a stimulator). In this study, we address three major issues of implantable wireless neural interface system.

The first issue is the low efficiency of the power transmission system and the limited power supply for an implanted device. Most implanted wireless devices are powered via either an inductive link or a non-rechargeable battery [6,9,10,12,13,14,15,16,17,18,19]. However, each system has its disadvantages: the former, a battery-free inductive link, requires very close distance between the implanted device and the external powering module, which restricts the movement of the subject wearing the device while providing low efficiency wireless power transmission; the latter, a non-rechargeable battery having its own life span, demands inevitable surgeries to replace an exhausted battery due to the limited life span.

The second issue concerns the way data communication is performed over long distances with low power consumption. Inductive, optical and radio links have been used to establish a data link between the implanted device and the external device [8,9,16,17,20,21]. However, inductive and optical links require patients to remain an uncomfortable position for proper data communication because data transmission and receiving devices should be kept in very close distance. Also, inductive-link-based medical communication system can be easily affected by other communication systems in the same frequency band [22]. Meanwhile, radio links in the industrial, scientific and medical (ISM) radio bands such as IEEE 802.15.1-Bluetooth and IEEE 802.15.4-ZigBee produce large losses above 500 MHz because electromagnetic energy is absorbed by the high water content in body tissues [23].

The third issue is to devise an electronic circuit to share an electrode between the stimulator and the amplifier. If a concurrent neural stimulation and recording is required, the experimenter needs two distinct devices, one as a stimulator and one as an amplifier and two different types of electrode, one for recording and the other for stimulation; this typically causes difficulties when inserting all devices and electrodes into the limited inner space of a body. Additionally, large stimulus-related artifacts can easily contaminate small recorded neural signal of interest [24,25,26,27,28,29].

To overcome the aforementioned issues, this study proposes a new implantable wireless neural interface system that consists of a wireless power consortium (WPC)-compliant power transmission circuit, a medical implant communication service (MICS)-band-based radio link and cuff-electrode path controllers.

First, the WPC-compliant power transmission circuit is able to wirelessly charge the rechargeable battery through transcutaneous wireless power transmission. This circuit can eliminate unnecessary battery replacements, guarantee a higher efficiency than conventional inductive power transmission systems and reduce battery-charging time.

Next, the MICS-band-based radio link provides longer wireless data communication range with smaller power consumption compared to the previous studies using inductive or optical link. The Federal Communication Commission (FCC) regulated the MICS band at 402-405 MHz for implantable biotelemetry applications. By using the MICS band, operating distance between the implanted device and external device was increased, which is not possible with short-range magnetic coupling or optical link. The 402–405 MHz frequency band has superior signal propagation characteristics, that is, at the MICS band the RF signal in the human body could be less attenuated than other bands [30]. Therefore, the MICS-band-based RF transceivers consume a smaller current compared to other solutions that use the ISM radio bands. Additionally, unexpected interference with other services can be prohibited with particularly designed the channel bandwidth, output power level and duty cycle [22].

Finally, the newly devised switching circuit for the cuff-electrode path controller allows concurrent nerve stimulation and recording with a single cuff electrode while reducing the number of cables, cuff electrodes and electronic devices required.

In this paper, we suggest a practical and affordable implantable wireless neural interface system for simultaneous neural signal recording and stimulation, which can be implemented using readily available small form factor commercial off-the-shelf (COTS) components, instead of an application-specific integrated circuit (ASIC) that requires high cost and design delays. The validity of the proposed design was confirmed by bench tests and in vivo experiments using rabbit models. 

## 2. Materials and Methods 

**Figure 1:**
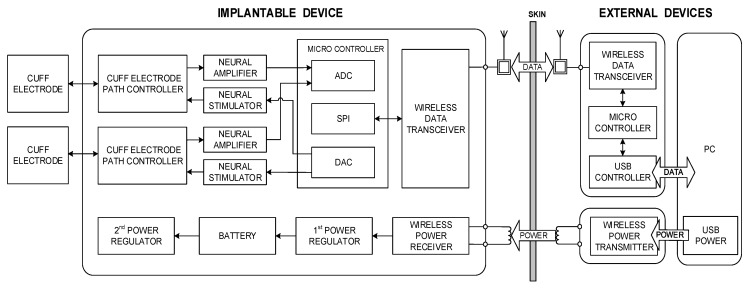
Block diagram of the proposed implantable wireless device system.

Figure 1 shows a conceptual block diagram of the proposed system, which includes an implantable device and associated external devices. The external devices wirelessly transmit commands and power to the implantable device. The implantable device stimulates peripheral nerves and records neural signals through the cuff electrodes; it also wirelessly transmits the device’s status and recorded neural signals to the external devices. In addition, a personal computer (PC)-based graphical user interface (GUI) was also developed which specifies the commands required for stimulation and recording as well as displays the stimulation waveforms and recorded data. The details of each part and in vivo experimental procedures are described in the following sections.

### 2.1. Neural Signal Interface

#### 2.1.1. Cuff Electrode

**Figure 2:**
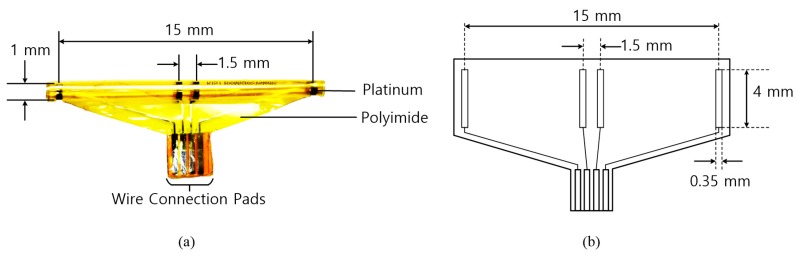
Nerve cuff electrode: (**a**) photographic image and (**b**) schematic image.

The cuff electrodes used in this research were developed by Center for BioMicroSystems at Korea Institute of Science and Technology (KIST) [31]. A revised quasi-tripolar configuration was adopted to alleviate an imbalance from tissue impedance inside the cuff. When two middle electrodes were placed at the center of the cuff electrode, a significant improvement in the signal-to-interference ratio was achieved for the EMG signals and the stimulus artifacts when compared to a conventional tripolar cuff [5]. To fabricate a cuff electrode, a polyimide substrate and platinum electrode contacts were used. The polyimide substrate was self-biased to curl into a roll with an inner diameter. As shown in Figure 2, the fabricated cuff electrode has the following major characteristics: 15 mm between the end electrodes, 1.5 mm between the middle electrodes and a 1-mm diameter. The diameter of the cuff electrode was designed to firmly wrap around the tibial and peroneal nerves of the rabbit and to avoid damage from excessive mechanical stresses on the nerve. Table 1 shows the physical characteristics of the fabricated cuff electrode.

#### 2.1.2. Neural Amplifier

**Figure 3:**
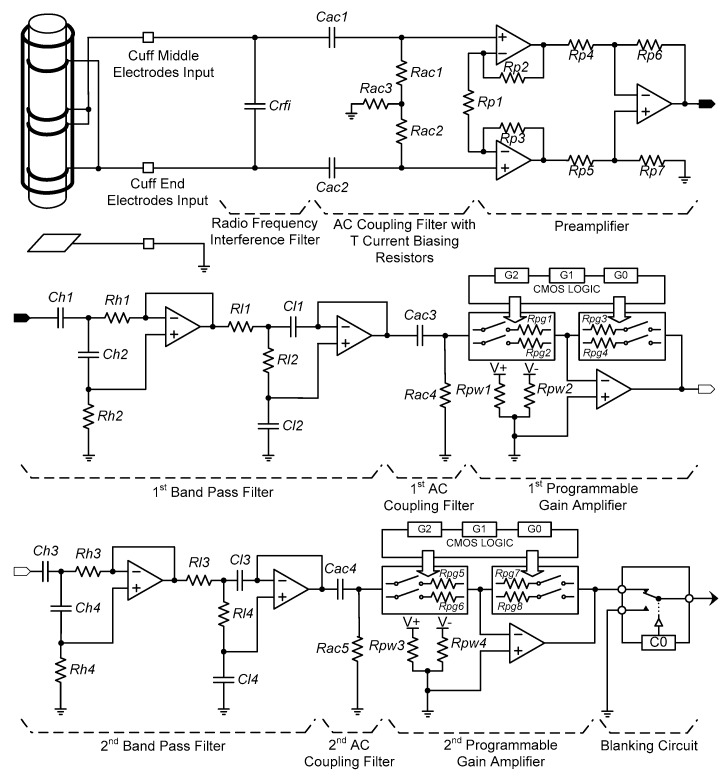
Block diagram of the proposed neural amplifier.

To acquire neural signals, the cuff electrode recording system developed in the previous study [5] was modified to be suitable for implantable device. The neural amplifier was configured with a radio frequency interference (RFI) filter, AC coupling filters, a preamplifier, band pass filters, programmable gain amplifiers (PGA) and a blanking circuit (see Figure 3).

A precision, low-noise instrumentation amplifier (INA118, Burr-Brown, Tucson, AZ, USA) was used as the preamplifier and its gain was fixed at 100. The first band pass filter and the first PGA followed the preamplifier. Next, the second band pass filter and the second PGA were connected. The band pass filters consisted of a second-order Butterworth high pass filter with a 300-Hz cut-off frequency in cascade with a second-order Butterworth low pass filter with a 5000-Hz cut-off frequency. Two dual matched PGAs (LTC6911, Linear Technology, Milpitas, CA, USA) were used to allow selecting various gains and each was located with an AC coupling filter at the output stage of the band pass filters.

The desired gain of each channel was adjusted using a three-bit parallel interface to select voltage gains. As a result, the overall amplifier system was designed to have an adjustable gain between 100 and 1,000,000 and a maximally flat magnitude response in the frequency range of 300 to 5000 Hz, where the energy spectrum of the electroneurogram (ENG) signal is dominantly distributed.

Electrical stimulation generates stimulus artefacts, evoked ENG and electromyogram (EMG) signals, which continue for about 10 ms after the electrical stimulation [5,24,25,26,27,28,29]. Therefore, a blanking circuit is required to prevent acquiring unwanted signals: the stimulus artefacts and evoked ENG and EMG signals [32]. In this study, single-pole, double-throw (SPDT) switches (ADG836L, Analog Devices, Norwood, MA, USA) were used to construct the blanking circuit. The operation time of the blanking circuit could be set by a 16-bit timer in a microprocessor (MSP430F1611, Texas Instruments, Dallas, TX, USA) from 0 to 10 ms with a resolution of 1 ms. During the selected period of time, the devised blanking circuit disconnected the amplified neural signal and tied the blanking circuit’s output with the ground so that only the signal of interest was acquired.

#### 2.1.3. Neural Stimulator

**Figure 4:**
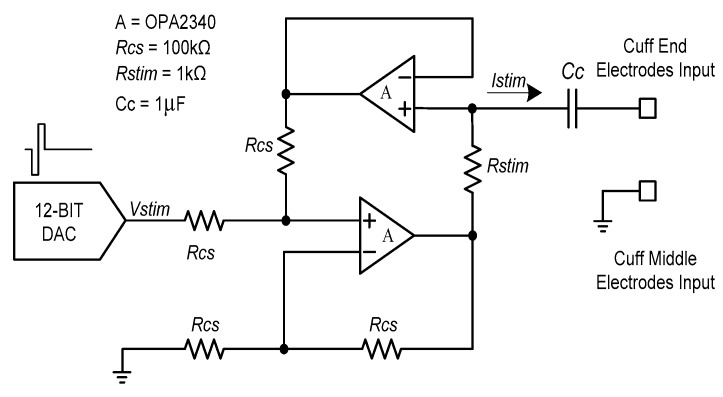
Block diagram of the proposed voltage-controlled current source.

Figure 4 shows the voltage-controlled current source (VCCS) that was used for neural stimulation. The current source should provide a sufficiently large output impedance and a minimized output current error should be guaranteed over a wide range of stimulation frequencies [33,34]. To maintain these conditions, a dual operational-amplifier current source was used as the current source. A 1µF coupling capacitor was used to prevent charge buildup at the electrode–tissue interface.

Also, symmetrical biphasic stimulation was used to prevent the tissue electrode damage [35,36,37]. The symmetrical biphasic pulses were generated via a digital-to-analogue converter (DAC) in the microprocessor and applied to the input of the VCCS. The generated biphasic pulses had a 20-ms period and each phase (i.e., the cathodal phase and the anodal phase) had a fixed duration of 200 µs. Therefore, the biphasic waveform with a fixed 50-Hz stimulation frequency was used throughout the neural stimulation procedure. Contrary to a fixed stimulation frequency and duration, the amplitude of the biphasic pulse could be set from 0 to 1500 µA in 20-µA increments. The stimulation current could be calculated using the following equation:

(1) $$Istim=\;\frac{Vstim}{Rstim}.$$

**Figure 5:**
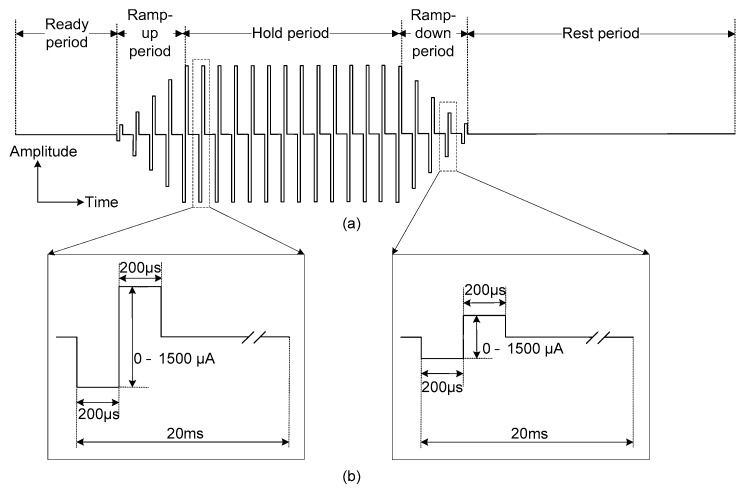
Stimulation waveform: (**a**) including ramp-up and ramp-down periods and (**b**) details of a single pulse.

During neural stimulation, ramp-up and ramp-down periods are essential to avoid sudden muscle contraction and relaxation, which may cause spastic muscle reactions [38]. To generate diverse stimulation waveforms with ramp-up and ramp-down periods, the stimulation waveform consisted of five different periods: a ready period, a ramp-up period, a hold period, a ramp-down period and a rest period. Each period could be set to have a duration of 20 to 2000 ms in 20-ms increments (see Figure 5).

#### 2.1.4. Cuff-Electrode Path Controller

A cuff-electrode path controller was designed to use one cuff electrode for recording and stimulation. The cuff electrode’s path was determined by two analogue switches (TS3A24159, Texas Instruments, Dallas, TX, USA).

**Figure 6:**
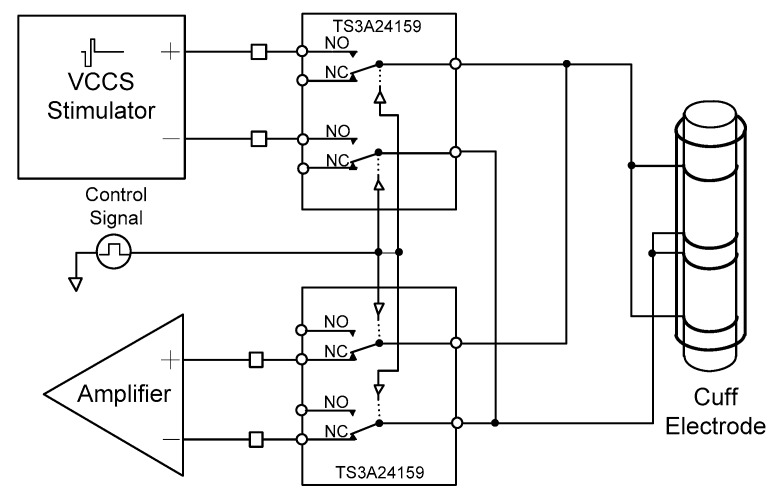
Block diagram of the proposed cuff-electrode path controller.

As shown in Figure 6, when the control signal of the cuff-electrode path controller was set to “low”, the cuff electrode began to work with the amplifier. Conversely, when the control signal of the cuff-electrode path controller was “high”, the cuff electrode began to work with the stimulator.

### 2.2. Wireless Data Communication

Two ZL70102 medical implant RF transceivers (Microsemi, Aliso Viejo, CA, USA) were used for the MICS-band-compliant wireless data communication. As an ultra-low-power device, the ZL70102 medical implant RF transceiver provides high-speed communication with raw data rates of 800, 400, or 200 kbps. The ZL70102 device has three different power-consuming stages: a sleep stage (100 nA), a wake-up stage (380 nA) and a stage of high-speed transmitting or receiving (5 mA) (ZL70102 Design Manual) [39]. Two microprocessors (MSP430F1611, Texas Instruments, Dallas, TX, USA) were used, one in the implantable device and one in the external device, to control each wireless data transceiver using the serial peripheral interface (SPI). For operation in the 400-MHz MICS band and the 2.45-GHz ISM band, a dual-band helical antenna for the external device and a wire whip antenna for the implantable device were used.

To establish a session between the implantable device and the external device, a 2.45-GHz wake-up transmitter in the external device broadcasted wake-up messages to search for the implantable device until it received a response from the implantable device. When the implantable device received a wake-up message from the external device and responded to the external device, wireless MICS-band link was established between the implantable device and the external device for wireless data transmission.

**Figure 7:**
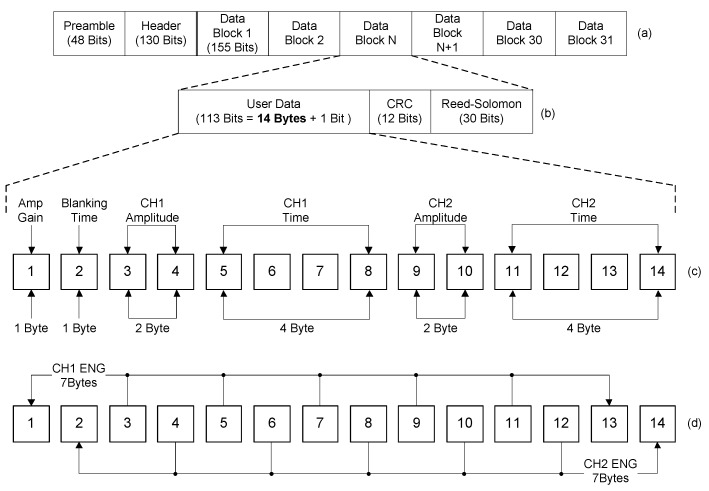
Block diagram of the proposed data packet and its structure: (**a**) data packet, (**b**) data block, (**c**) user data section for commands and (**d**) user data section for acquired neural signals.

Figure 7 shows the block structure of the data packet for the wireless data transceiver used in this study. Both wireless data transceivers in the implantable device and the external device used the same data packet, which was comprised of 31 blocks and the user data section consisted of 14 bytes. By using the given user data sections, the external device sent commands and the implantable device transmitted the acquired neural signals. Figure 7c shows the user data section, organized for commands; this data section carried parameters for the neural amplifier gain, the blanking time, the stimulation amplitude and the stimulation period.

The amplified ENG signals were digitized with a sampling rate of 10 kHz per channel by an internal analogue-to-digital converter (ADC) in the microprocessor and were then packaged for transmission. Figure 7d shows the user data section designed for the acquired ENG signals. The user data section included two channels of neural signals. Within the 14 bytes, the odd bytes contained one channel of neural signals and the even bytes contained the other channel of neural signals. The 31 blocks of user data sections including recorded neural signals were accumulated into a data packet and the data packet was sent from the implanted device to external device every 43.4 ms.

### 2.3. Wireless Power Transmission

Inefficient wireless power transmission devices waste energy and take a long time to charge their rechargeable batteries. To minimize these weaknesses as well as to provide reliable and transcutaneous wireless power transmission, Texas Instruments’ high efficiency wireless power transferring solution, known as Qi wireless charging, satisfying WPC standard was used. The WPC developed an open interface standard that defines wireless power transfer using inductive charging. Also, recent study regarding wireless power transmission for implantable devices revealed that the induced magnetic field using Qi wireless charging was well below the limits set by ISO 14117 evaluating the electromagnetic compatibility (EMC) of active implantable cardiovascular devices [40].

An integrated wireless power supply receiver (BQ51013, Texas Instruments, Dallas, TX, USA) was used to receive power and a wireless power controller (BQ500211, Texas Instruments, Dallas, TX, USA) was used to transmit power. The BQ51013 is a WPC-compliant, integrated, receiver IC for wireless power transfer in portable applications. It has a small package (1.9 mm × 3 mm) that is suitable for use in a compact implantable device. In addition, the BQ500211 controls to transmit external power to the implantable device wirelessly. To save energy, the power transmitter stops supplying power by entering a low-power standby mode when there is no need for power. The transmitter and receiver communicate with each other. For communication, various types of communication packets are defined, such as identification and authentication packets, error packets, control packets, end power packets and power usage packets [41]. Unlike other inductive power transmission devices, WPC-compliant devices can avoid certain dangerous situations such as unintended energy transfer to incompatible devices and reaching unacceptable temperature levels in the power transferring device caused by metal objects by using identification communications and a metal object detection function [42,43]. Therefore, users can reduce the time required for charging a rechargeable battery in the implantable device and improve safety by limiting wireless power transmission when there are some undesirable situations.

Two different coils were fabricated for wireless power transmission. Unlike the wireless power transmitting coil detailed in the standards, certain customizations were required for the wireless power receiving coil to be fitted into the small form factor of the implantable device. The receiving coil was designed to be as large as possible to maximize the overlapped area with the transmitting coil while also ensuring that it could be embedded in the implantable device. These customizations could have included a larger-diameter wire, the use of bifilar wire, or a printed circuit board (PCB) coil; for this design, however, bifilar wire was selected to fabricate the wireless power-receiving coil because it could provide a higher efficiency in the limited area than the larger-diameter wire or a PCB coil [42]. As a result, the number of turns in the wireless power receiving coil was set to 13 with an outer diameter of 23.6 mm. As a shielding material, a ferrite plate with a 2-mm thickness was attached to the bottom of the wireless power receiving coil to minimize the degradation of efficiency and prevent direct heat damage to susceptible components behind the wireless power receiving coil [42]. Other characteristics of the wireless power transmission coils are shown in Table 2.

### 2.4. In Vivo Experiments

**Figure 8:**
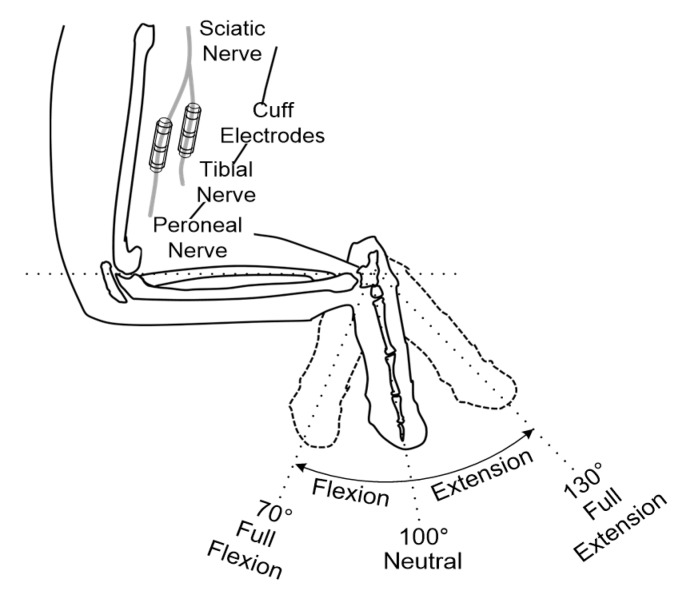
Implantation sites of the cuff electrodes and definition of the ankle joint angles.

**Figure 9:**
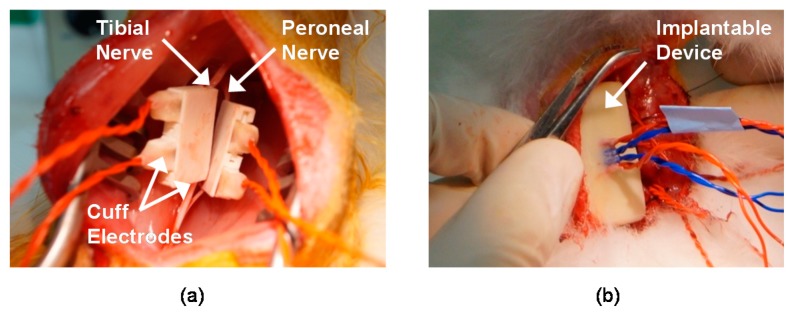
Surgical procedure for implant placement: (**a**) cuff electrodes wrapped around the tibial and peroneal nerves, (**b**) implantable device inserted under the back skin of a rabbit.

In vivo experiments were performed using adult male New Zealand white rabbits (2–3 kg) to record and stimulate the peripheral nerves. Throughout the experiments, the rabbits were kept and handled in accordance with the regulations of the Institutional Animal Care and Use Committee of Korea Institute of Science and Technology (KIST). An anesthetic mixed with Zoletil 50 (Virbac, Carros, France) and Rompun (Bayer, Seoul, Korea) was used for anesthesia. After removing the hair on the thigh of the animal, approximately an 3 cm incision on the rear side of the left hind limb was made to expose the sciatic nerve through the avascular fascicular junction [44]. Through the incision, the tibial and peroneal nerves were gently exposed; then, the two nerves were carefully separated to ensure space for the installation of the two cuff electrodes (see Figure 8). Finally, the cuff electrodes, pre-soaked in saline, were wrapped around the tibial and peroneal nerves, respectively (see Figure 9a).

Another incision was made on the back skin of the rabbit to insert the implantable device (see Figure 9b). After inserting the implantable device under the back skin, all incision sites were closed. To remove all voluntary and reflex responses, the rabbit was under anesthesia during experiments. Supplemental doses of the anesthetic were administered every 20 min throughout the surgery and the experiments. During the experiments, the rabbit was placed on an apparatus and its ankle was fastened on a rotatable manipulator.

Two apparatuses were devised for the in vivo experiments: one was equipped with a high-torque gear servomotor (HS-7954SH, Hitec, Shoeburyness, UK) to consistently provide passive movements of the rabbit’s ankle in the sagittal plane; the other was designed to stably support the evoked movements of the rabbit’s ankle via electrical stimulation that was generated from the implantable device. The angle of the rabbit’s ankle joint was measured using a goniometer (SG65, Biometrics), a power and amplifier module (Electro Goniometer Interface Box, Motion Lab Systems, Baton Rouge, LA, USA) and a data acquisition card (NI PCI-6034E, National Instruments, Austin, TX, USA).

#### 2.4.1. Neural Signal Recording

To evaluate the recording performance of the proposed system in the in vivo experiments, the prior studies [45,46] were modified as follows. A series of identical ramp-and-hold, flexion-extension movements were used to passively rotate the rabbit’s ankle joint using a high-torque gear servomotor. Full- flexion, neutral and full-extension positions were set at 70°, 100° and 130° (see Figure 8) and each position had a holding time of 2, 0.5 and 2 s, respectively. An ankle excursion of 60° was performed from full-extension to full-flexion and vice versa. A constant angular velocity of 60°/s was maintained during the movements. The ENG signals were acquired with a gain of 100 dB and the blanking time was set to 0 ms. All parameters for generating electrical stimulation were set to 0 to only record muscle afferent activities caused by passive ankle motions without the electrical stimulation.

#### 2.4.2. Simultaneous Neural Signal Recording and Stimulation

Another in vivo experiment was designed to evaluate the comprehensive performance of simultaneous recording and stimulation. In this experiment, bipolar pulses with a 400-µs duration, 20-ms period and 300-µA amplitude were used for tibial nerve stimulation and bipolar pulses with a 400-µs duration, 20-ms period and 500-µA amplitude were used for peroneal nerve stimulation. The ENG signals were acquired with a gain of 100 dB and the same time periods were applied to the both stimulation waveforms for the tibial and peroneal nerves: ready (1000 ms), ramp-up (200 ms), hold (1000 ms), ramp-down (200 ms) and rest (1500 ms) periods. Then, based on previous studies [5,24,25,26,27,28,29,47], the blanking time was set to 8 ms to prevent stimulus artefacts, evoked ENG and EMG signals caused by electrical stimulation. The ENG signals were recorded starting from the neutral position of the ankle angle at 100°.

## 3. Results

### 3.1. Neural Signal Interface

#### 3.1.1. Neural Amplifier

**Figure 10:**
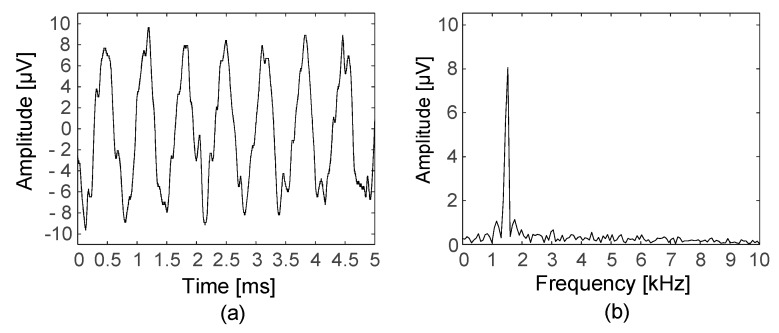
Measured output signal: (**a**) in the time domain and (**b**) in the frequency domain.

An evaluation circuit studied in [5] was used to simulate the differential and common-mode voltages as inputs to the neural amplifier. To verify the performance of the devised neural amplifier, the amplified output and frequency response of the overall amplifier system were measured. A sinusoidal signal was provided with a 1-V peak amplitude and 1.5-kHz frequency and was attenuated to the level of a 10-µV peak; this attenuated differential voltage represented the ENG signal. The common-mode voltage was assumed to be the power-line interference. A sinusoidal signal was provided with a 1-V peak amplitude and a 60-Hz frequency without attenuation. Figure 10 shows the output signal of the neural amplifier in the time domain and the frequency domain with test signals generated by the evaluation circuit. The 10-µV, 1.5-kHz differential voltage was amplified and the 1-V, 60-Hz, common-mode voltage was filtered out.

**Figure 11:**
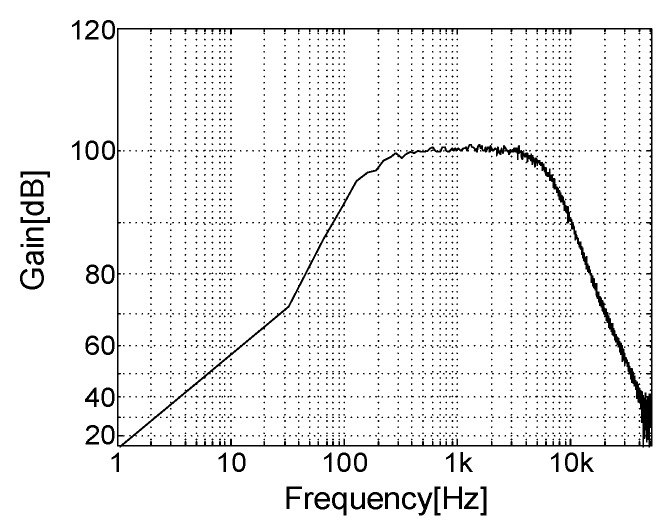
Frequency response of the overall amplifier system.

As shown in Figure 11, the frequency response was measured with a dynamic signal analyser (35670A, Agilent, Santa Clara, CA, USA). The −3 dB bandwidth was found to be between 237–5478 Hz. These results were obtained when the neural amplifier’s total gain was 100 dB.

**Figure 12:**
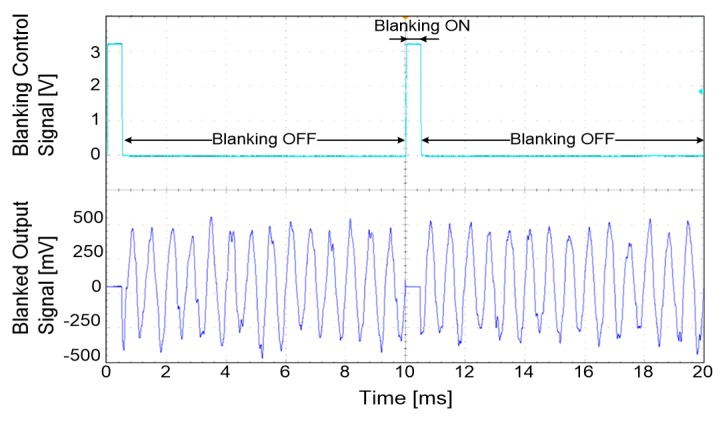
Measured output signal blanked during a 500-ms control signal.

The blanking circuit was used to selectively record signals so that it could prevent acquiring unwanted signals. The performance of the blanking circuit was evaluated with the same signal used for the verification of the neural amplifier and the blanking control signal was generated by a function generator with a duration of 500 µs. As shown in Figure 12, the output signal was grounded when the blanking control signal was in its ON state. Table 3 shows the summarized characteristics of the neural amplifier.

#### 3.1.2. Neural Stimulator

To evaluate the performance of the devised VCCS, a 1-kΩ load resistor was connected to the output of the VCCS. The output current of the VCCS was converted to a voltage across the load resistor and the load resistor voltage was measured and compared to the VCCS input voltage.

**Figure 13:**
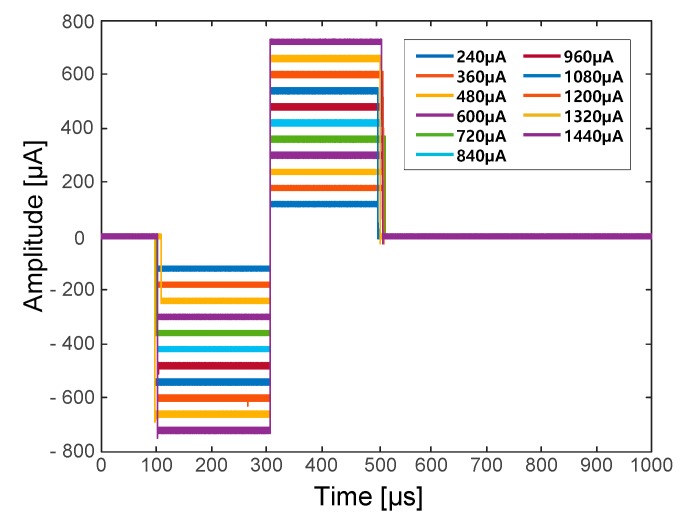
Converted VCCS output current waveforms with a 1 kΩ load resistor.

Figure 13 shows the converted current stimulation waveforms through the load resistor with various input voltages generated from the DAC of the microprocessor. It was possible to generate precise biphasic, constant-current pulses for stimulation with minimal errors. Table 4 shows the summarized characteristics of the neural stimulator.

#### 3.1.3. Cuff-electrode Path Controller

Two SPDT analogue switches (TS3A24159, Texas Instruments, Dallas, TX, USA) were used to implement the cuff-electrode path controller. To evaluate the function of the cuff-electrode path controller, conductivity and output signals were measured. For the conductivity test, the resistance between the inputs and outputs was measured while changing the control signal of the cuff-electrode path controller. Approximately 0 Ω was measured for each connection when the inputs and outputs were connected by the control signals.

**Figure 14:**
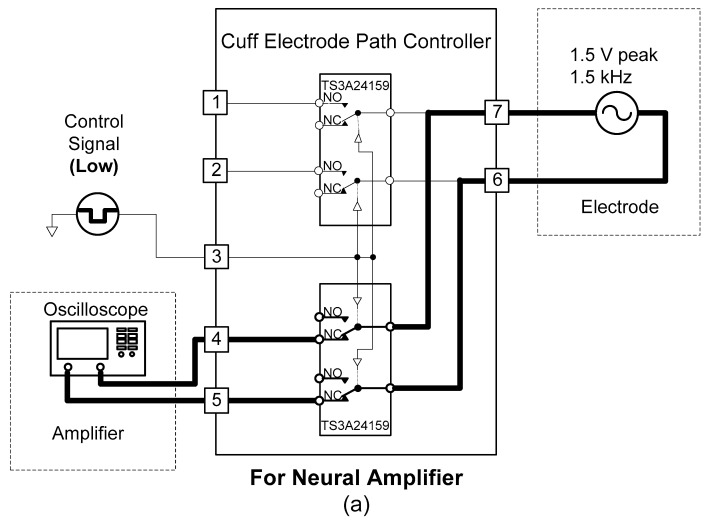

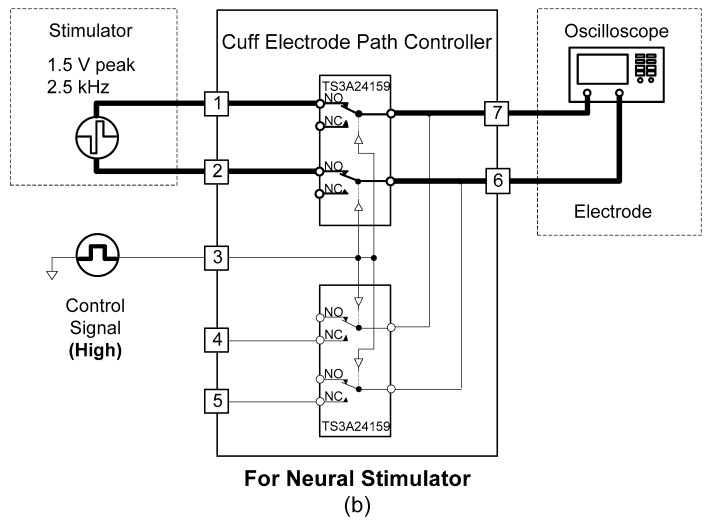
Circuit connections used to evaluate the cuff-electrode path controller: (**a**) for neural amplifier and (**b**) for neural stimulator.

Next, the output characteristics of the cuff-electrode path controller were verified with the circuit connections shown in Figure 14 while changing the control signal. When the cuff-electrode path controller was working with the neural amplifier with a control signal of “low”, a 1.5-kHz, 1.5-V peak, sinusoidal signal was connected between pins 7 and 6 and the output signal was measured between pins 4 and 5 (see Figure 14a). Conversely, when the cuff-electrode path controller was working with the neural stimulator with a control signal of “high”, the biphasic pulses were connected between pins 1 and 2 and the output signal was measured between pins 7 and 6 (see Figure 14b). From the experimental results, it was confirmed that the cuff-electrode path was successfully shared between the neural amplifier and stimulator based on the control signal.

#### 3.1.4. Stimulation Waveforms and Control Signals

**Figure 15:**
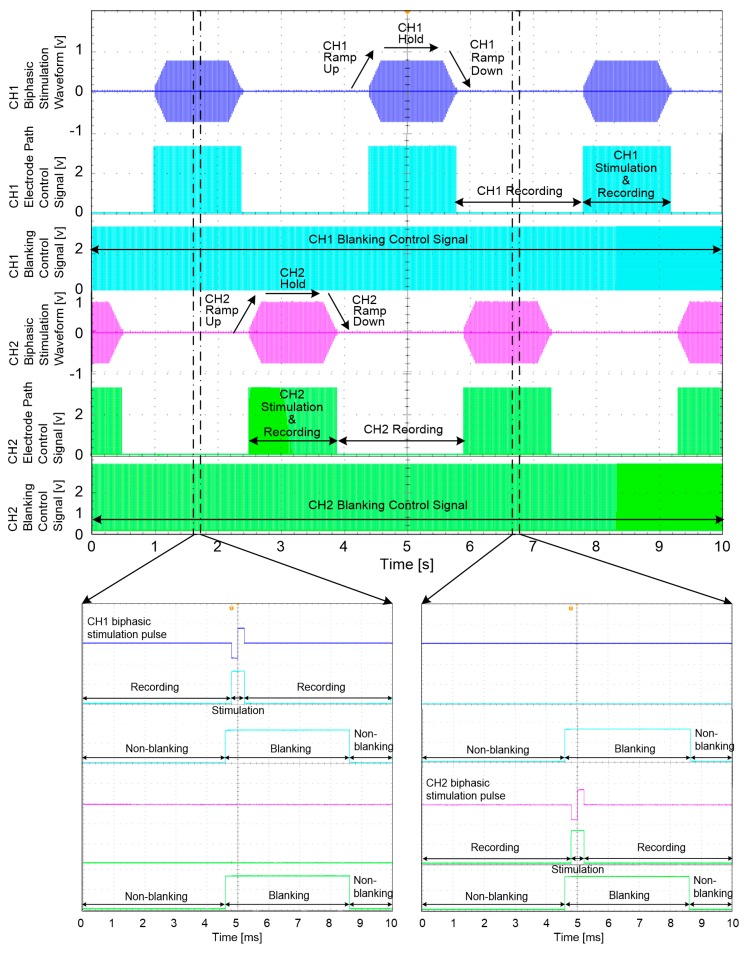
Stimulation waveforms and control signals for the cuff-electrode path controllers and blanking circuits.

Figure 15 shows an example of the generated stimulation waveforms for the VCCS inputs and the control signals for the electrode path controllers and blanking circuits. These signals were measured from the output ports of the microprocessor and their parameters were wirelessly specified by the designed GUI software and external device.

The upper portion of Figure 15 shows the signals measured over 10 s. The lower portions of Figure 15 show enlargements of the upper portion of Figure 15, where all signals were captured synchronously with a single biphasic stimulation pulse. In this example, to stimulate the rabbit’s nerve, biphasic stimulation waveforms including ramp-up and ramp-down periods were produced and each pulse in the biphasic stimulation waveform had a 400-µs duration. The same duration was applied to the electrode path control signals to allow the cuff electrodes to only work with the stimulator during this time period and to work with the amplifier during the other time period. As shown in the lower portion of Figure 15, the electrode path control signal had the same 20-ms period as the biphasic stimulation pulse and maintained as “high” for the fixed duration of 400 µs when only the biphasic stimulation pulse was generated. Each blanking control signals began from the start of the biphasic stimulation pulse and was maintained for 4 ms. Although the blanking control signals can prevent stimulus artefacts, evoked ENG and EMG signals from affecting one channel, these unwanted signals still remain on the opposite channel. Therefore, an additional blanking control signal was also applied to the opposite channel at the same time with the same duration.

### 3.2. Wireless Data Communication

To evaluate the reliable maximum distance of the wireless data communication, sinusoidal signals were applied to the implantable device and were monitored through the devised GUI program while increasing the distance between the implantable device and the external device. The implantable device was placed under subcutaneous tissue after hermetic packaging. During this experiment, it was determined whether the received data were identical to those applied to the implantable device. The established session was maintained at up to 1.98 m and the sinusoidal signals were acquired without distortion. The current consumption of the MICS-band-based RF transceiver was measured by using a current probe (TCP0030A, Tektronix, Beaverton, OR, USA) in different modes: data receiving mode (4.7 mA) and data transmitting mode (5.6 mA). The power consumption of the RF transceiver in Table 5 was calculated when 3.3 V was supplied. Other characteristics for the wireless data communication are shown in Table 5.

### 3.3. Wireless Power Transmission

To verify the performance of the devised wireless power transmission function, its efficiency and maximum operating distance were investigated. The efficiency of the wireless power transmission system was calculated using the ratio of the output power to the input power. Next, the maximum distance was measured by increasing the distance of the implantable device and the external wireless power transmission module until there was no wireless power transmission. The implantable device was placed under subcutaneous tissue. From the outside, the external wireless power transmitting module was positioned close to the implantable device and the output power (transmitting power) and the input power (receiving power) were measured to calculate the efficiency of the devised wireless power transmission. The efficiency of the configured wireless power transmission device was 67% and the wireless power receiving and transmitting procedures were found to operate up to a maximum distance of 11 mm. Table 6 lists the characteristics of the wireless power transmission.

### 3.4. Assembled Implantable Device

**Figure 16:**
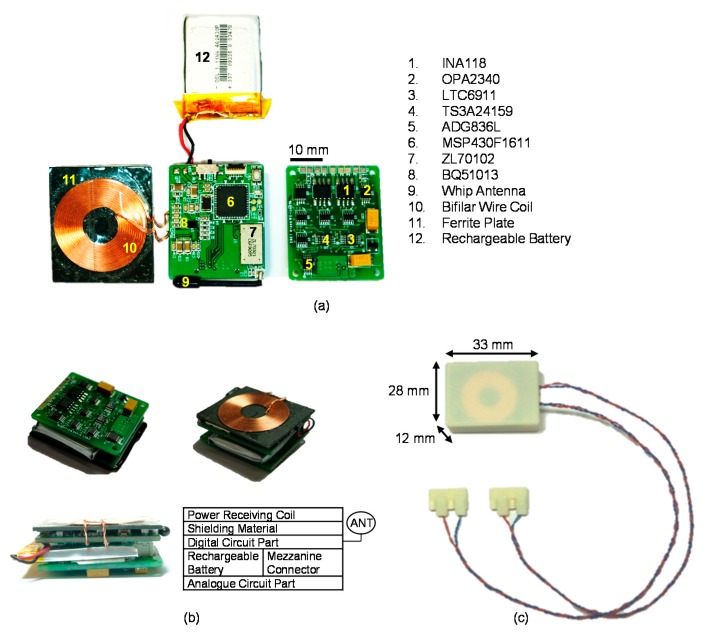
Photograph of the implantable device: (**a**) disassembled view and key components of the implantable device, (**b**) assembled view and (**c**) encapsulated implantable device and cuff electrodes by bio-compatible PolyJet photopolymer (MED610).

Figure 16a shows the disassembled implantable device. The implantable device was implemented in two distinct parts: an analogue circuit for the neural amplifier and stimulator and a digital circuit for wireless data communication and wireless power receiving. The analogue and digital circuits were intentionally separated to protect the analogue paths from digital interference and power-supply noise. As shown in Figure 16b, the complete implantable device was assembled in a layered manner by stacking the analogue circuit, rechargeable battery, digital circuit with its antenna, shielding material and power receiving coil. For packaging the implantable device and two cuff electrodes, the bio-compatible PolyJet photopolymer (MED610) that is a rigid medical rapid prototyping material was used. After packaging the assembled implantable device with MED610, we coated the device with dimethylsiloxane (PDMS, sylgard 184, DowCorning, Midland, MI, USA) for in vivo experiments. The base and the curing agent were mixed in 10 to 1 mass ratio. A vacuum chamber was used to eliminate bubbles for 20 min. The curing process was conducted for 1.5 h in a pre-heated oven at 60 °C. The cuff electrodes were encapsulated by closures. In the proposed system, a stable electrical connection and strain relief were accomplished by means of a closure that enveloped the cuff electrodes, lead wires and nerve bundle. The closures were comprised of a base plate and slider plate of the rigid medical rapid prototyping material. To verify the hermetically sealed implantable device, the implantable device was immersed in 0.9% saline for a week. During this time, wireless data communication and wireless power transmission were established once a day. The disabled devices in the test were not implanted for animal experiments. Figure 16c shows the encapsulated implantable device, which was 28 mm × 33 mm × 12 mm and 21 g. The implantable device consumed 6 mA in the ready stage, 25 mA during the only-recording stage and 33 mA during the simultaneous-recording-and-stimulation stage. The operating times of the implantable device were expected to be 185 h (ready), 44.4 h (recording only) and 33.6 h (recording and stimulation) when the fully charged battery (3.7 V, 1.11 Wh) was used without additional charging (see Table 7).

### 3.5. In Vivo Experiments

#### 3.5.1. Performance of Neural Signal Recording

**Figure 17:**
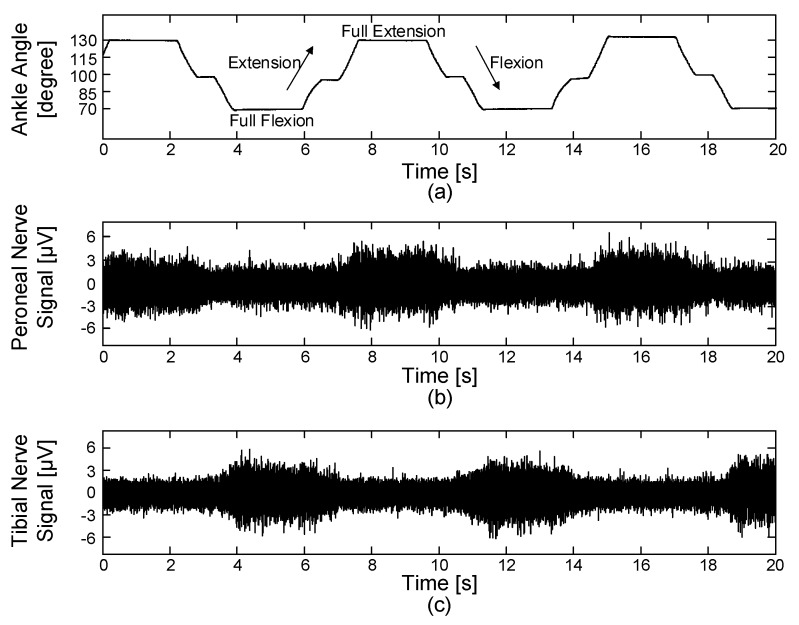
Acquired joint angle and ENG signals during the passive movements of the rabbit’s ankle: (**a**) joint angle, (**b**) ENG signals from the peroneal nerve and (**c**) ENG signals from the tibial nerve.

Figure 17 shows the recorded joint angle and ENG signals during the passive movements of the rabbit’s ankle. The muscle afferent activity from the tibial nerve showed the highest amplitude at full-flexion (70°). Conversely, the muscle afferent activity from the peroneal nerve showed the highest amplitude at full-extension (130°). The ENG signals were properly amplified and filtered with a high signal-to-noise ratio of 5.46 dB and were then wirelessly transmitted at 10 kHz sampling rate from the implantable device to the external device. The recorded ENG signals from both peroneal and tibial nerves corresponded to the results obtained in the previous studies [44,45,46]. During neural signal recording with the passive movement of the rabbit’s ankle, the stimulation path is completely disconnected by the cuff-electrode path controller. The obtained neural signals were monitored and saved using the devised GUI. The result showed that the proposed implantable wireless neural interface system could successfully record muscle afferent activities during ramp-and-hold, flexion-extension movements.

#### 3.5.2. Performance of Simultaneous Neural Signal Recording and Stimulation

**Figure 18:**
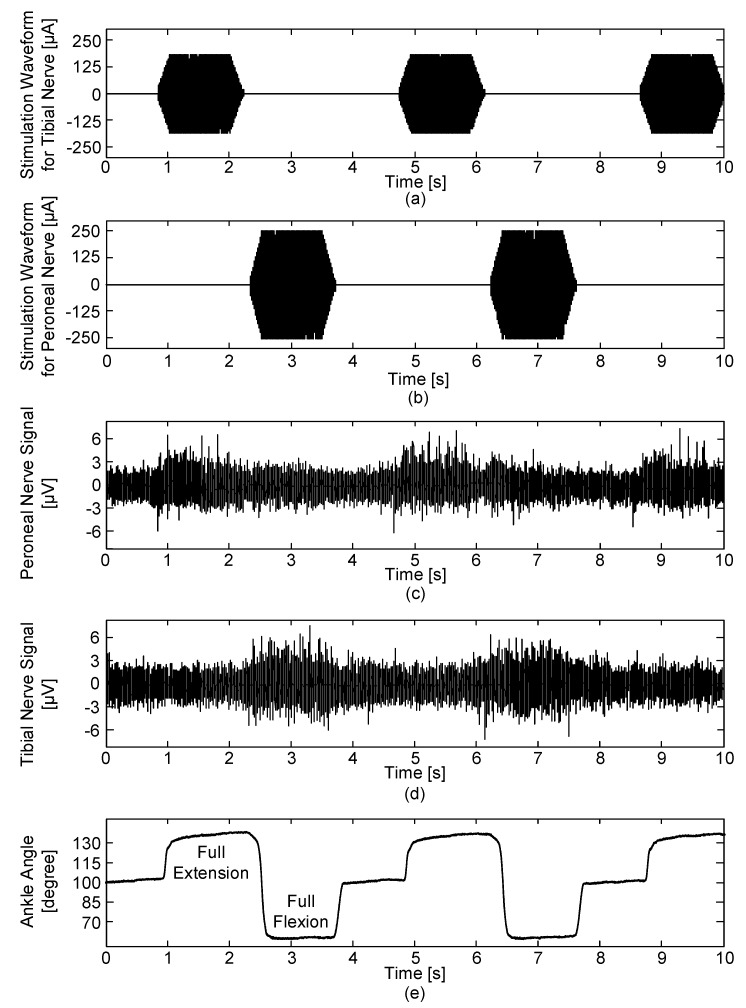
Acquired joint angle and ENG signals during the electrical stimulation period: (**a**) biphasic stimulation waveforms for the tibial nerve, (**b**) biphasic stimulation waveforms for the peroneal nerve, (**c**) ENG signals from the peroneal nerve, (**d**) ENG signals from the tibial nerve and (**e**) joint angle.

For neural signal recording with the electrical stimulation, the neural stimulator was only connected to the cuff electrode during electrical stimulation period by the cuff-electrode path controller. The obtained neural signals were monitored and saved using the devised GUI. As shown in Figure 18, when electrical stimulation was applied to the peroneal nerve, the dorsi-flexor muscles were activated and the ankle was rotated to the full-flexion position; strong ENG signals were recorded from the opposite tibial nerve when the ankle reached full-flexion. On the other hand, when electrical stimulation was applied to the tibial nerve, the plantar-flexor muscles were activated and the ankle was rotated to the full-extension position; strong ENG signals were recorded from the opposite peroneal nerve when the ankle reached full-extension. Otherwise (i.e., when there was no stimulation), the ankle moved to the neutral position without showing dominant ENG signals from either the tibial or peroneal nerve. As shown in Figure 18e, there was no undershoot, overshoot, or ringing and the rabbit’s ankle showed mild movements by the delicate biphasic stimulation waveforms including ramp-up and ramp-down periods that were intentionally designed to prevent sudden muscle contraction and relaxation. The devised implantable device worked as designed for concurrent neural signal recording and stimulation. The cuff-electrode path controller made it possible to use one cuff electrode for both recording and stimulation and the blanking circuit properly blocked the interference of stimulus artefacts, evoked ENG and EMG signals caused by the electrical stimulations.

#### 3.5.3. Performance of Blanking Circuit during Simultaneous Neural Signal Recording and Stimulation

**Figure 19:**
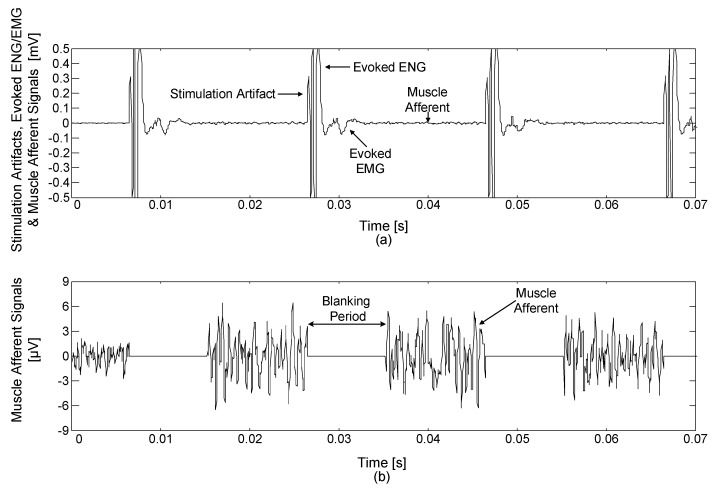
Acquired neural signals: (**a**) when the blanking processes were not applied and (**b**) when the blanking processes were applied.

An additional experiment was conducted to evaluate the performance of the blanking circuit. Figure 19a shows the acquired neural signals including stimulation artifacts, evoked ENG signals, evoked EMG signals and muscle afferent signals. This data recording was performed with 40-dB gain without using the blanking circuit. The stimulation artifacts, evoked ENG and EMG signals appeared in synchrony with 50-Hz electrical stimulation, which interfered with the recording of muscle afferent signals. However, this issue could be avoided by using the devised blanking circuit. Figure 19b shows the obtained muscle afferent signals when the blanking circuit was applied to eliminate the interference. This data recording was performed with 100-dB gain and a synchronized blanking pulse with each stimulus switched the neural signal to ground for a duration of 8 ms. By using blanking circuit, the neural signals of interest could be recorded without the interference from the electrical stimulation.

## 4. Discussion

In this study, we proposed a novel implantable wireless neural interface system, which can be rapidly implemented and modified with cost effective COTS components. The neural interface system includes a WPC-compliant power transmission circuit, an MICS-band-based radio link and a cuff-electrode path controller for simultaneous neural signal recording and stimulation using a single cuff electrode. The maximum reliable operating distance for wireless power transmission was found to be approximately 11 mm and the overall efficiency was measured to be 67%, which is higher than conventional wireless power transmission devices [7,9,10,48,49]. In addition, the wireless data communication guaranteed a sufficient data rate of 400 kbps while consuming low power (receiving: 4.7 mA, transmitting: 5.6 mA) and providing a longer data transmission range of 1.98 m compared to devices that use inductive or optical communications [8,9,11,19,50]. The cuff-electrode path controller made it possible to use one cuff electrode at the same implant site for both neural signal recording and stimulation, enabling the cuff electrode path to be shared for the neural amplifier and stimulator based on its control signal. The devised system allowed the experimenter to select a wide range of stimulation and recording parameters. Through the GUI software, the neural stimulation and recording parameters could be wirelessly set and the acquired neural signals could be displayed in real-time and archived using a bidirectional MICS-band-based radio link.

Table 8 provides a comparison of the system presented in this paper with other systems published in recent years, including various elements: the numbers of neural signal recording channel and stimulation channel, gain, sampling rate, data communication, power supply, target area, weight and size. It is noted that we have limited the comparison to the studies that implemented wireless neural interface devices using COTS components only. The functionality of the previous studies [9,12,49] is restricted while solely working as a neural signal recorder or a neural stimulator. Hence, extra devices are required for the more sophisticated experiments where concurrent neural stimulation and recording are required. The neural interface devices introduced in [51,52] are equipped with both functions of neural recording and neural stimulation by using separated modules. However, it is difficult to implant the separated modules with a large size into the limited inner space of a body. Moreover, individual power sources, power regulating components and control processors are needed for each module. Contrary to these, the proposed system was integrated into one compact module allowing a simple insertion procedure. One compact implantable module has advantage in the aspect of aesthetic since it occupies small area without noticing by other people. Therefore, one compact module is desirable for implantable devices. Extra surgeries should be refrained as much as possible but using limited power sources [9,12,49,51,52,53,54] such as non-rechargeable batteries cannot avoid extra surgeries to replace the exhausted batteries with new one. To prevent extra surgeries, inductive power transmission and ultrasonic power transmission technologies are being used for supplying power to the implanted devices without rechargeable batteries. However, these methods highly restrict patients’ movement because they require very close contact to operate. To solve these issues, extra surgeries and limited mobility, we combined two strategies, the WPT compliant inductive power transmission and the rechargeable battery. Although patients’ movement is temporary limited during charging procedure, the patients can freely move after the charging procedure. Also, there is no need for extra surgeries to change an exhausted battery. Establishing an implantable data communication is possible by other ISM bands [9,12,49,51,52,53,54] but they are not as qualified as the MICS band for implantable medical devices. Furthermore, they are prone to establish vulnerable data communication and consume higher power when compared to the MICS band. The MICS band consumes smaller power than other radio bands to communicate between the implantable device and the external device since the attenuation of the RF signal is smaller in comparison with other bands in the human body. The ZL70102 MICS band RF transceiver used in this study consumes about 5 mA, while the other RF transceivers in Table 8 consume higher current: TX3A (7.5 mA), TX2 (12 mA), nRF24L01 (12 mA) and CC2500 (13 mA). The MICS band using the frequency range at 402–405 MHz is exclusively allocated for implantable biotelemetry applications by the FCC. Therefore, unexpected interference can be avoided with specifically designed channel bandwidth, output power level and duty cycle. Using the MICS band allowed the devised system to establish a robust, secure and low-power consuming wireless data communication link.

## 5. Conclusions

The results obtained during bench tests and animal experiments clearly showed that the devised implantable wireless neural interface system could simultaneously record neural activities and stimulate nerves while sharing a single cuff electrode. Furthermore, the devised system not only includes various functions: a neural amplifier, a neural stimulator, a cuff path controller, a blanking circuit, a wireless data transceiver, a wireless power receiver but also allows adjusting various recording and stimulation parameters: the gain of a neural amplifier, the stimulation periods, the stimulation amplitudes and the number of biphasic stimulation waveforms avoiding sudden muscle contraction and relaxation. Thus, the system proposed in this study is highly versatile and can be easily reconfigured for various implantable medical devices by their own purpose, especially such as closed-loop control based implantable neural prostheses requiring neural signal recording and stimulation at the same time. Although the animals used in this study were anesthetized to assess the developed system, further studies will be performed with freely moving animals by using the devised system for many potential applications in neuroscience requiring nerve stimulation and/or neural signal recording. In addition, more research on secure hermetic packaging and heating issues should be studied for long term implantation.

## Figures and Tables

**Table 1:** Physical characteristics of the nerve cuff electrode.

Parameter	Value
Diameter of cuff	1 mm
Thickness of cuff	18 µm
Distance between end electrodes	15 mm
Distance between middle electrodes	1.5 mm
Length of electrode	4 mm
Width of electrode	0.35 mm
Impedance @ 1 kHz	1.2 kΩ
Charge delivery capacity	22 µC/mm^2^

**Table 2:** Characteristics of the wireless power transmission coils.

Parameter	Value
**Wireless power transmitting coil**	
Outer diameter	43 mm
Inner diameter	20 mm
Thickness	2.1 mm
Turns	10
Layers	2
Strands	105
Inductance	24.12 µH
**Wireless power receiving coil**	
Outer diameter	23.6 mm
Inner diameter	9.8 mm
Thickness	0.3 mm
Turns	13
Layer	1
Strands	2
Inductance	6.04 µH

**Table 3:** Electrical characteristics of the neural amplifier.

Parameter	Value
Number of channels	2
−3 dB bandwidth	237–5478 Hz
Gain	38–123 dB
Noise @300 Hz~5 kHz, Gain 100 dB	792.47 nV
Sampling rate	10 kHz
Blanking time	0–10 ms

**Table 4:** Electrical characteristics of the neural stimulator.

Parameter	Value
Number of channels	2
Amplitude of biphasic pulse	0–1500 µA
Duration of cathodal and anodal phases	200 µs
Period of biphasic pulse	20 ms
Stimulation waveform	ready, ramp-up, hold, ramp-down, rest

**Table 5:** Characteristics of the wireless data communication.

Parameter	Value
Radio frequency	402–405 MHz (MICS band)
Data rate	400 kbps
Modulation	FSK, bidirectional
Power Consumption	15.51 mW (Receiving mode) 18.48 mW (Transmission mode)
Operating distance	~1.98 m

**Table 6:** Characteristics of the wireless power transmission.

Parameter	Value
Wireless power solution	WPC V1.1
Switching frequency	110–205 kHz
Input power of wireless power transmitter	3.73 W (5 V, 0.746 A)
Output power of wireless power receiver	2.5 W (5 V, 0.5 A)
Efficiency	67%
Operating distance	~11 mm

**Table 7:** Characteristics of the assembled implantable device.

Parameter	Value
Power consumption	19.9 mW (ready)
	82.5 mW (recording only)
	108.9 mW (stimulation and recording)
Battery Operational hours after fully charged	185 h (ready)
	44.4 h (recording only)
	33.6 h (stimulation and recording)
Battery charging time	1–1.2 h
Total dimension	28 mm × 33 mm × 12 mm
Total weight	21 g

**Table 8:** Comparison of wireless neural interface systems.

	This Work	Liang et al. [9]	Xu et al. [49]	Zhou et al. [12]	Ativanichayaphong et al. [53]	Liu et al. [51]	Pinnell et al. [54]	Zuo et al. [52]
**Recording Channels**	2	1	0	0	1	4	4	1
**Stimulation Channels**	2	0	2	1	1	6	2	1
**Gain**	40–120 dB	100 dB	-	-	84 dB	54–96 dB	34 dB	73 dB
**Sampling Rate**	10 kSPS	11 kSPS	-	-	10 kSPS	30 kSPS	500 SPS	10 kSPS
**Data Communication (Band)**	ZL70102 (MICS)	Inductive Link	CC2500 (ISM), Inductive Link	CC2500 (ISM)	TX3A, RX2A (ISM)	nRF24L01 (ISM)	CC2500 (ISM)	nRF24L01 (ISM)
**Power Supply**	WPT, Rechargeable battery	Inductive coupling	Inductive coupling	Battery	Battery	Battery	Battery	Battery
**Target Area**	Tibial nerve, Peroneal nerve	Sciatic nerve	Lumbar-sacral segment	Lumber-sacral segment	Dorsal horn, Periaqueductal gray, Anterior cingulate cortex	Brain	Brain	Dorsal horn, Periaqueductal gray
**Weight**	21 g	-	3.78 g	12.6 g	20 g	-	8.5 g	12 g (STIM), 15 g (REC)
**Size**	33 × 28 × 12 mm	45 × 30 × 12 mm	22 × 23 × 7 mm	33 × 24 × 8 mm	25 × 50 × 27 mm	56 × 36 × 15 mm (REC), 43 × 27 × 8 mm (STIM), 31 × 13 × 8 mm (BSN)	28 × 17 × 7 mm	27 × 8 mm (REC), 27 × 12 mm (STIM)
